# Case Report: Pediatric pontine abscess and ecthyma gangrenosum due to *Pseudomonas aeruginosa* septicemia

**DOI:** 10.3389/fped.2025.1449357

**Published:** 2025-01-29

**Authors:** Jonathan Theros, Madison Wolfe, Larry Kociolek, Irini N. Kolaitis

**Affiliations:** ^1^ The Feinberg School of Medicine, Northwestern University, Chicago, IL, United States; ^2^ Ann & Robert H. Lurie Children’s Hospital of Chicago, Chicago, IL, United States

**Keywords:** brainstem abscess, pseudomonas, ecthyma gangrenonsum, pontine abscess, immunodeficiency, sepsis, neurosurgery, infectious disease

## Abstract

Brainstem abscesses are remarkably rare, with only a few reports in the pediatric literature. Their presence portends high morbidity and mortality and most commonly present in the setting of immunodeficiency. A 5-year-old boy with a history of recurrent acute otitis media presented to the emergency department with rash, otorrhea, confusion, and fever. He was found to be in septic shock secondary to *Pseudomonas aeruginosa* bacteremia; a skin exam revealed multifocal ecthyma gangrenosum. He was initially treated with intravenous ceftazidime. Despite adequate antibiotic coverage he had persistent fevers. Whole-body magnetic resonance imaging revealed an expansile pontine mass; dedicated neuroimaging confirmed a 10 mm pontine abscess. Given the lack of neurological deficits on examination, he was treated non-operatively with intravenous cefepime for 9 weeks followed by oral levofloxacin for 30 days and made a nearly complete clinical recovery. Extensive immunodeficiency workup did not identify an immunologic defect. Prompt action through interdisciplinary care meetings and avoidance of early diagnostic closure resulted in an excellent neurological outcome for this patient with this rare case of a *P. aeruginosa* brainstem abscess.

## Introduction

Brain abscesses are remarkably rare in the general population, with an incidence of less than 1 in 100,000 per year (1), and approximately less than 1% of brain abscesses are found in the brainstem (2, 3). There have only been a couple dozen reports of brainstem abscesses in the pediatric literature, none that implicate *Pseudomonas aeruginosa* as the causative pathogen (4, 5). They are most commonly located in the pons followed by the midbrain (6, 7). The most common pathogens in children are *Staphylococcus* spp., *Streptococcus* spp., and *Listeria* spp. (2, 4, 5, 8). Brainstem abscesses are associated with high morbidity and mortality and typically associated with various immunodeficiencies (2, 3, 9).

Brainstem abscesses often present with fever, headache, focal neurological deficits, and, less commonly, seizures (3, 4). Treatment is dependent on clinical features, typically consisting of a combination of intravenous antibiotics, aspiration via craniotomy, and/or burr-hole stereotactic aspiration (3, 4). Brainstem abscesses have been successfully treated with antibiotic therapy alone with favorable outcomes (5).

*P. aeruginosa* is a versatile pathogen that causes a wide variety of infectious pathologies, including otitis externa, folliculitis, pneumonia, septic arthritis, and urinary tract infections among others, and can widely disseminate causing bacteremia and sepsis (10). Ecthyma gangrenosum (EG) is a rare presentation of *P. aeruginosa*, with the majority of cases occurring in immunocompromised hosts (11). EG is characterized by painless papules that evolve into pustules and eventually a black eschar; approximately three-quarters of cases are caused by *P. aeruginosa* (11). The presence of EG in the setting of sepsis and neutropenia carries a mortality rate as high as 77% (12).

We report a case of a likely immunocompetent boy who developed pseudomonal sepsis and ecthyma gangrenosum who was ultimately found to have a pontine abscess. This case report was constructed in reference to the CARE guidelines (13) and the clinical course is summarized in Table 1.

**Table 1:** Summary of key events in clinical course.

Day	Event
0	Initial symptom onset: fever, ear pain
	Urgent care: started on amoxicillin–clavulanic acid for bilateral acute otitis media
3	Presented to ED: septic shock, ecthyma gangrenosum, positive blood cultures for *P. aeruginosa*
	Admitted to ICU: intubated, vasopressors, empiric vancomycin and cefepime
7	Transitioned to ceftazidime, extubated, afebrile, transferred to floor
11	Fever, concern for occult infectious source
	Transitioned back to cefepime
13–14	Whole-body MRI demonstrated expansile pontine mass
	Dedicated brain MRI shows 10 mm pontine abscess
15	Swallow study revealed silent aspiration, nasogastric tube placed
	Neurological examination otherwise normal
20	Repeat MRI demonstrated mild improvement
22	Discharged home with peripherally inserted central catheter on cefepime
33–48	Immunodeficiency workup unrevealing
69–71	MRI brain with residual findings, peripherally inserted central catheter and nasogastric tube removed
	Transitioned to oral 30-day course of levofloxacin

## Case report

A 5-year-old boy initially presented to an urgent care facility for evaluation of fever and ear pain and was prescribed amoxicillin–clavulanic acid for bilateral acute otitis media. On day 3 after symptom onset, he presented to the emergency department with a fever, rash, altered mental status, sore throat, and ear pain. His medical history was notable only for recurrent acute otitis media and bilateral tympanostomy tube placement 4 years prior that had self-extruded 6 months before presentation. His perinatal care was unremarkable, and he was up to date on all recommended vaccinations. He lived at home with his mother, father, and grandfather, attended pre-kindergarten, and had no recent travel. He was born in the United States and his family history was non-contributory.

**Figure 1:**
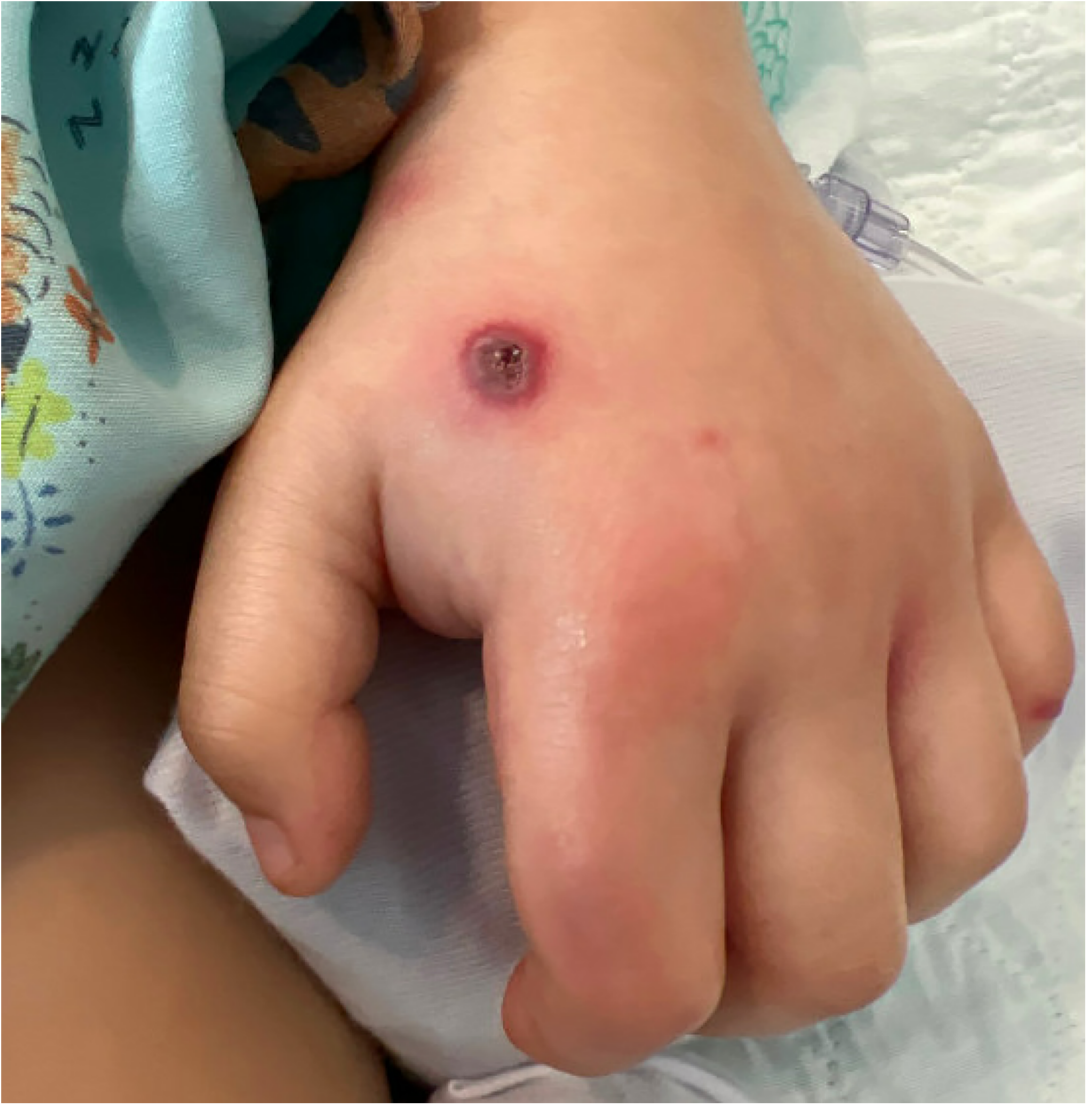
Ecthyma gangrenosum rash on left hand on day 3.

The patient’s examination on presentation was notable for a Glasgow Coma Scale (GCS) score of 13, fever, hypotension, otorrhea, and a necrotic rash on his trunk, genitals, face, and extremities (Figure 1). He underwent endotracheal intubation and was admitted to the intensive care unit (ICU) for treatment of uncompensated septic shock, where he received intravenous vasopressors and empiric vancomycin and cefepime. Blood cultures and biopsy of skin lesions both grew *P. aeruginosa*, confirming the diagnosis of EG. Labs were also notable for the detection of adenovirus and coronavirus HKU1 from a multiplex PCR assay performed on a nasopharyngeal swab, as well as leukopenia (white blood cell count: 2.89 thou/μl), absolute lymphopenia (723 lymphocytes/μl), borderline neutropenia (lowest absolute neutrophil count: 1.70 thou/μl), thrombocytopenia (platelet count: 43 thou/ml), and anemia (hemoglobin: 10.6 g/dl). Assays for von Willebrand factor activity and ADAMTS13 factor ruled out thrombotic thrombocytopenic purpura. Computed tomography (CT) of the brain on day 3 of symptoms (first day of admission) showed mild opacification of the left middle ear and mastoid with no signs of brain involvement. There was no clear evidence of tympanic membrane perforation on physical examination.

**Figure 2:**
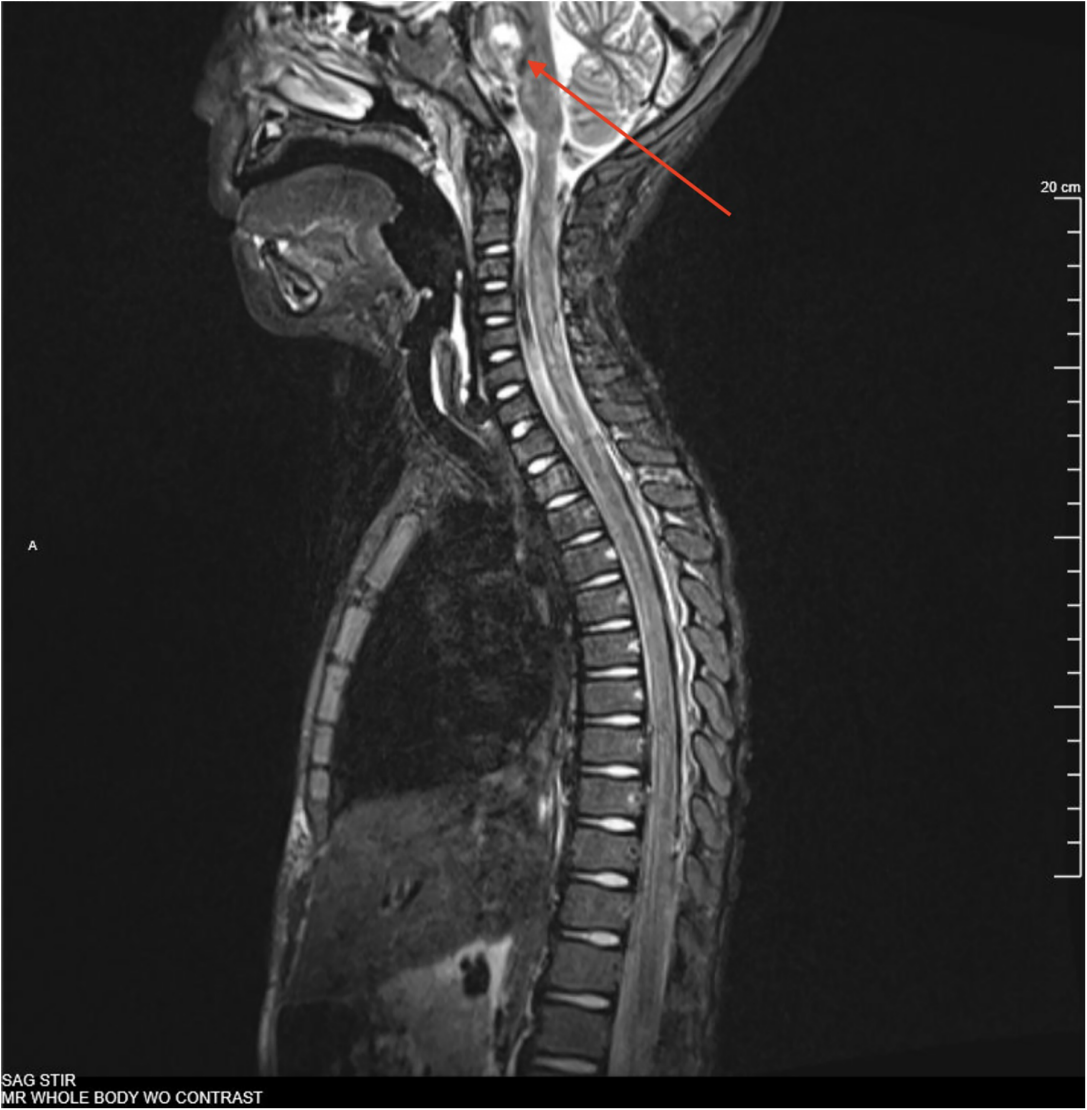
Full-body MRI, sagittal, STIR showing 9 mm STIR hyperintense expansile pontine mass on day 13.

**Figure 3:**
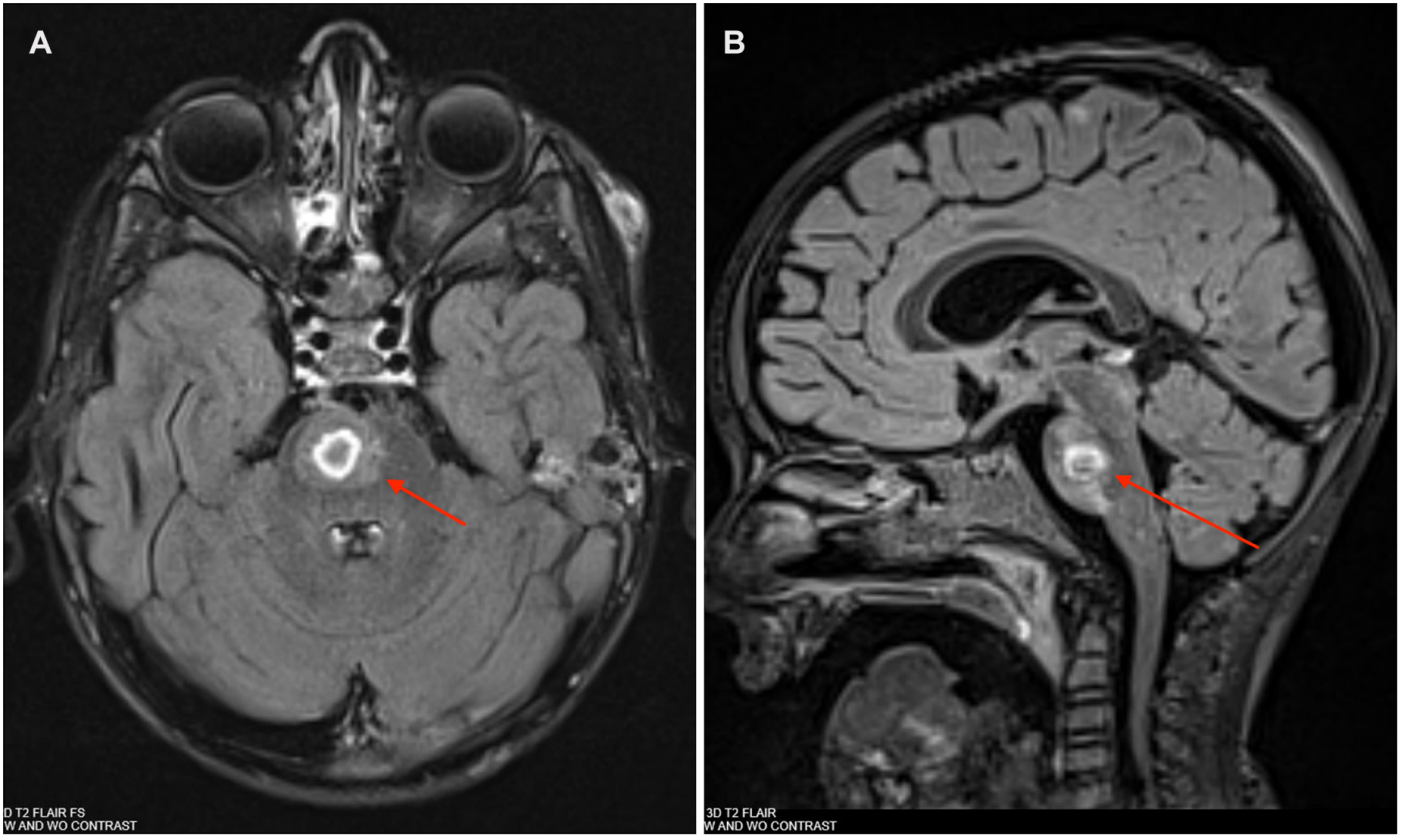
**(A)** Brain MRI: T2 post-contrast axial Fluid-attenuated inversion recovery (FLAIR) showing a 10 mm × 9 mm × 9 mm abscess in right paramedian pons on day 14. **(B)** Brain MRI: T2 post-contrast sagittal FLAIR showing a 10 mm × 9 mm × 9 mm abscess in R paramedian pons on day 14.

On day 7, the boy was transitioned to ceftazidime based on culture sensitivities. Topical mupirocin was used to prevent super-infection of EG lesions. He was afebrile, extubated, and able to transfer to the floor. On day 11, he began to report arthralgia and malaise and developed fevers again. The examination was notable for significant generalized weakness thought to be secondary to deconditioning. Antibiotic therapy was changed to cefepime, given the borderline susceptibility of the pseudomonal isolate to ceftazidime [minimum inhibitory concentration (MIC) was 4 μg/ml]. Given the patient’s high risk for occult infection and lack of localizing symptoms, on day 13, a whole-body magnetic resonance imaging (MRI) was completed that revealed a 9 mm expansile, short tau inversion recovery (STIR) hyperintense lesion in the pons along with diffuse skin micro-abscesses consistent with EG (Figure 2). Follow-up dedicated brain MRI showed a 10 mm lesion involving the right paramedian pons with peripheral ring enhancement and a central area of restricted diffusion, consistent with an abscess (Figures 3A, B). In addition, there were punctate-enhancing foci along the periphery of the cerebral hemispheres representing likely septic emboli. Echocardiography with bubble study was negative. The neurosurgical team did not recommend surgical intervention given his stable clinical condition. His seizure risk was determined to be low and no anti-convulsant medications were started.

On day 15, the patient began coughing with oral intake, and a swallow study showed thin liquid aspiration with laryngeal penetration. The neurological examination remained stable with no obvious cranial nerve or focal deficits, and a nasogastric tube was placed with dysphagia though to be secondary to deconditioning. Repeat brain MRI on day 20 showed stable to mildly improved findings. On day 22, he was discharged on continued intravenous cefepime therapy and a nasogastric tube for thin fluids.

**Figure 4:**
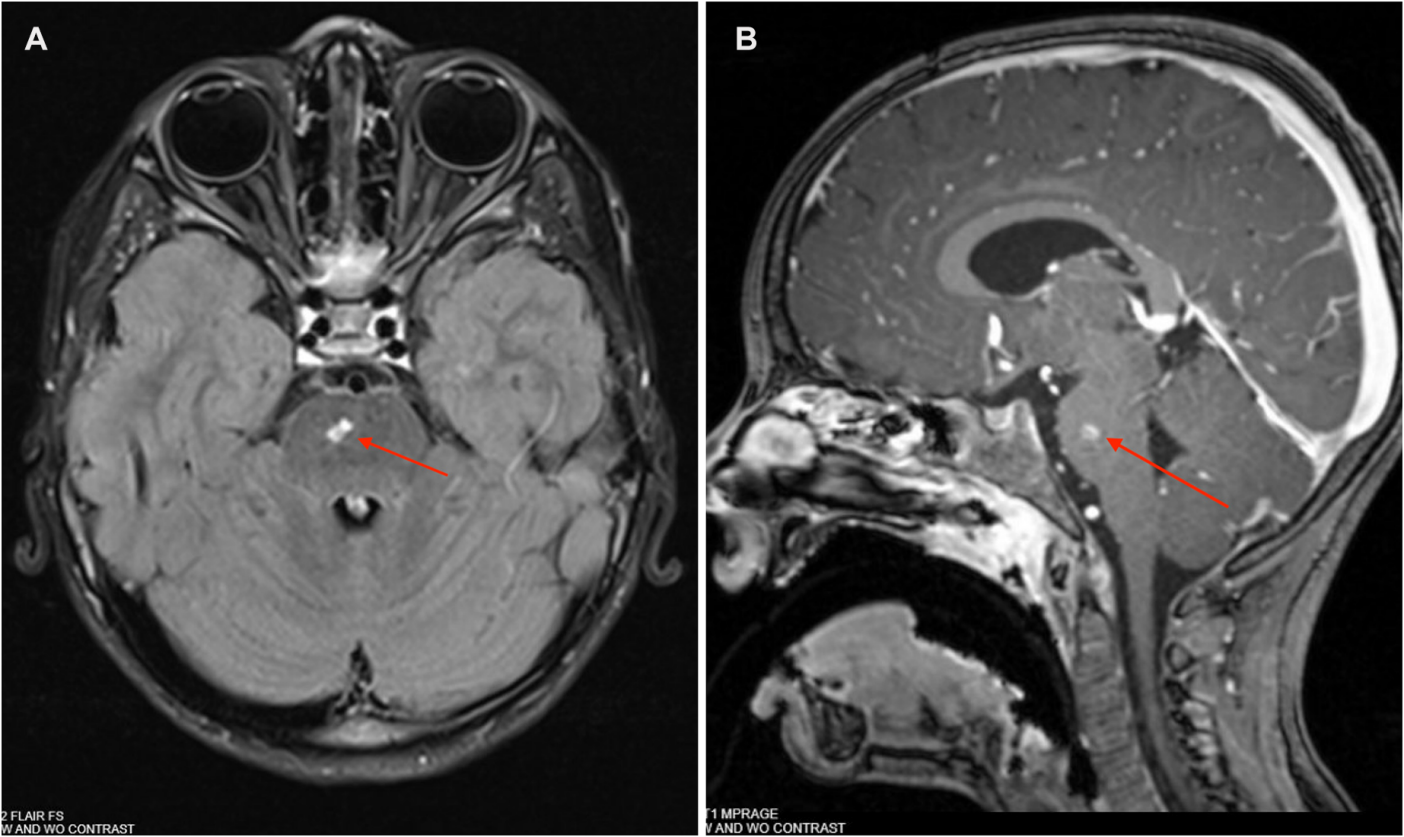
**(A)** Brain MRI: T2 post-contrast axial FLAIR showing a 5 mm × 3 mm abscess in right paramedian pons on day 69. **(B)** Brain MRI: T1 post-contrast sagittal MPRAGE showing a 5 mm × 3 mm abscess in R paramedian pons on day 69.

On day 42, an outpatient MRI examination showed a decreased size of the pontine lesion to 6 mm in its greatest dimension and near resolution of the central diffusion restriction. MRI on day 69 of the clinical course showed mild residual signal changes and enhancement (Figures 4A,B). On day 71, cefepime (50 mg/kg/dose every 8 h) was discontinued after approximately 9 weeks of therapy; the patient was started on levofloxacin (10 mg/kg daily) for a 30-day course; his peripherally inserted central catheter and nasogastric tube were removed; and he returned to school after approximately 8 weeks of physical therapy. There were no adverse reactions or complications noted 6 months after his hospitalization, and he did not have recurrent otitis media.

An immunologic evaluation on day 3 included negative leukemia/lymphoma immunophenotyping flow cytometry, negative human immunodeficiency virus (HIV) serology, negative chronic granulomatous disease flow cytometry dihydrofolate reductase assay, low total complement (13 mg/dl), low CD3+ cell count (792 mm^3^), low CD3+ CD4+ cell count (371 mm^3^), low NK cell count (91 mm^3^), low IgG (427 mg/dl), low normal IgM (55.4 mg/dl), and normal IgA (164 mg/dl). No vaccinal titers were completed. Labs were repeated at various time points after day 30 and all perturbations normalized or rebounded. The genetic immunodeficiency panel was negative for known homozygous pathologic variants, although there were seven heterozygous sequencing variants of uncertain significance including one in each of C5 (Arg1214Lys), ICAM1 (Pro219Arg), TBX2 (Asp161Glu), TERT (Ala288Val), TICAM1 (Arg71Gln), UNG (6232A > G), and ZAP7 (Ala378Val), and two heterozygous copy number variants (duplication exons 2–3) on the *RHOH* gene. This assay was performed using next-generation sequencing and assessed in accordance with the American College of Medical Genetics and Genomics (ACMG) guidelines (14).

## Discussion

We describe a case of a presumed immunocompetent 5-year-old child who developed septicemia, EG, and a pontine abscess caused by *P. aeruginosa.* To the best of our knowledge, this is one of the first pediatric cases of a brainstem abscess from *P. aeruginosa* described in the literature. The boy was successfully treated with intravenous and oral antibiotics and made a full recovery with little to no residual clinical deficits and without the need for operative intervention.

Brain abscesses may result from hematogenous spread or via contiguous spread possibly due to retrograde venous transport from a structure such as the ear (6, 15). *Pseudomonas* spp. have been reported as possible pathogens for brain abscesses in the setting of a middle ear infection (9), and the presence of aural purulent discharge is present in approximately 10% of patients with brain abscesses (3). However, given concurrent bacteremia, EG, and the rare location in the brainstem, our patient's abscess likely arose as a result of hematogenous seeding. The mechanism of EG includes bacterial invasion of the media and adventitia of vessels, particularly the arterioles and venules (16). It is possible that a similar mechanism of invasion occurred in the vascular supply of the pons.

Given the severity of the patient’s clinical presentation, including EG, which presents in an immunocompromised host in the majority of cases (11), an underlying immunodeficiency was strongly suspected. However, extensive workup for an immunodeficiency did not identify a known immunodeficiency. Notably, several variants of unknown significance were identified on his genetic immunodeficiency panel. However, given the lack of a personal history of prior serious infections or family history of immunodeficiency, suspicion of an unidentified immunodeficiency remains low at this time.

The lack of obvious cranial nerve involvement is surprising given the findings of an approximately 1 cm pontine lesion on neuroimaging. The patient's dysphagia represents a possible manifestation of cranial nerve involvement, although the location of cranial nerve IX and X nuclei does not correlate with the pontine lesion. The follow-up examination on day 65 showed an asymmetric palate raise with uvula deviation to the right, indicating a possible left-sided cranial nerve X palsy that may have been obscured on examinations earlier in the course due to intubation and examination limitations in the setting of acute illness. An alternate explanation for his dysphagia is deconditioning from an ICU stay and 4-day intubation.

Brainstem abscesses have been successfully treated with antibiotic therapy alone with favorable outcomes (5). Given this patient’s lack of clear neurologic deficits and peripheral cultures identifying *Pseudomonas*, the highly likely pathogen in the brainstem, it was determined that surgical intervention was not warranted. The management of this patient included a prolonged course of intravenous and oral anti-pseudomonal antibiotics only.

A critical component of this case was the decision to complete a whole-body MRI examination after recrudescence of fever on day 10, in addition to the switch from ceftazidime back to cefepime given the borderline susceptibility to ceftazidime (MIC 4 μg/ml). A full-body MRI examination was performed owing to the high probability of occult infection given the patient's clinically apparent superficial micro-abscesses. More commonly employed modalities, such as chest radiographs, were considered; however, given the patient's generalized malaise, clinical worsening, and lack of localizing symptoms, it was determined to be definitive and comprehensive imaging was warranted. This approach is limited to high-resource settings where MRI is readily available. These decisions were made with consultation to multidisciplinary teams who specialize in patients with sepsis and multi-organ dysfunction syndrome-associated immune dysregulation and were key to reopening the differential diagnosis throughout the course.

In summary, this case highlights the importance of multidisciplinary care and the avoidance of early diagnostic closure. In carefully chosen cases, brainstem abscesses can be treated with medical therapy alone, without neurosurgical intervention, and lead to excellent outcomes.

## Data Availability

The original contributions presented in the study are included in the article/Supplementary Material, further inquiries can be directed to the corresponding author.
